# Mesenchymal Stem Cells in Inflammation Microenvironment Accelerates Hepatocellular Carcinoma Metastasis by Inducing Epithelial-Mesenchymal Transition

**DOI:** 10.1371/journal.pone.0043272

**Published:** 2012-08-28

**Authors:** Yingying Jing, Zhipeng Han, Yan Liu, Kai Sun, Shanshan Zhang, Guocheng Jiang, Rong Li, Lu Gao, Xue Zhao, Dong Wu, Xiong Cai, Mengchao Wu, Lixin Wei

**Affiliations:** 1 Tumor Immunology and Gene Therapy Center, Eastern Hepatobiliary Surgery Hospital, Second Military Medical University, Shanghai, People’s Republic of China; 2 Department of Combined Treatment, Eastern Hepatobiliary Surgery Hospital, Second Military Medical University, Shanghai, People’s Republic of China; The University of Hong Kong, China

## Abstract

In response to inflammation, mesenchymal stem cells (MSCs) are known to migrate to tissue injury sites to participate in immune modulation, tissue remodeling and wound healing. Tumors apply persistent mechanical and pathological stress to tissues and causes continual infiltration of MSCs. Here, we demonstrate that MSCs promote human hepatocellular carcinoma (HCC) metastasis under the influence of inflammation. The metastasis promoting effect could be imitated with the supernatant of MSCs pretreated with IFNγ and TNFα. Interestingly, treatment of HCC cells with the supernatant leads to epithelial-mesenchymal transition (EMT), an effect related to the production of TGFβ by cytokines stimulated MSCs. Importantly, the levels of MSCs expressing SSEA4 in clinical HCC samples significantly correlated with poor prognosis of HCC, and EMT of HCC was strongly associated with a shorter cancer-free interval (CFI) and a worse overall survival (OS). Therefore, our results suggest that MSCs in tumor inflammatory microenvironment could promote tumor metastasis through TGFβ-induced EMT.

## Introduction

Mesenchymal stem cells (MSCs) can differentiate into multiple lineages such as chondrocytes, osteocytes, adipocytes, myocytes, and astrocytes, which is a potential candidate of stem cells for cellular and genetic therapy [1], [2]. MSCs are found in the bone marrow and have also been isolated from other sites in the body such as adipose tissue and uterus [3]. The phenotype of MSCs is identified by the absence of the CD34 and CD45 hematopoietic cell markers and is positive for CD90 and CD105 [4]. MSCs can be expanded more than 10^4^-fold in culture without loss of their multilineage differentiation potential. Because of these properties, MSCs have been exploited for their potential to repair or regenerate damaged tissues of mesenchymal origin, including tendon repair, cartilage regeneration, and support of engraftment after chemotherapy [5], [6].

Inflammation is an essential part of the malignant microenvironment [7]. Chemokines, leukocyte infiltration, and cytokines are crucial elements, which contribute to cancer-related inflammation. Attracted by chemokines, a variety of mesenchymal stem cells (MSCs) from the bone marrow are recruited at injury sites in a number of pathological conditions such as inflammation, tissue repair and also neoplasia [8], where they may become myofibroblasts or tumour-associated macrophages (TAF) [9], and play a role in facilitating hepatoma progression [10]. Exposure to mutiple inflammatory factors in the local microenvironment, such as interferon-γ (IFNγ), tumor necrosis factor-α (TNFα), and Interleukin-1 (IL-1), MSCs secrete several cytokines like Interleukin-10 (IL-10) [11], transforming growth factor-β (TGFβ) [11], [12], hepatocyte growth factor (HGF) [12], and vascular endothelial growth factor (VEGF) [13], which promote immunosuppression, angiogenesis and tumor growth. Although MSCs have been evaluated in clinical phase I and II studies for immunomodulation therapy after liver transplantation [14], the potential effect of MSCs on HCC metastasis in inflammatory microenvironment is still poorly understood.

Epithelial-mesenchymal transition (EMT) is considered as an important feature of physiological condition. Recently, studies have revealed that similar but pathological transitions occur during the progression of cirrhosis, endowing epithelial cells with mesenchymal features [15]. Some results also indicate that both invasion and metastasis may be critically associated with EMT, which is a key event in the tumor invasion process whereby epithelial cell layers lose polarity together with cell-cell contacts and then undergo a dramatic remodeling of the cytoskeleton [16]. In addition, EMT also helps cancer cells disruption of cell-cell adherence, loss of apico-basal polarity, matrix remodeling, increased motility and invasiveness [17]–[19], which has important clinical significance in HCC metastasis. Current study suggests that MSCs actively recruited to tumor stromal microenvironment may act as a stimulus to induce EMT in breast cancer cells and actively increase breast cancer metastatic potential [17]. However, whether MSCs in tumor inflammatory microenvironment stimulated may lead tumor metastasis by inducing EMT of HCC cells, as well as the clinicopathologic characteristics of MSCs in HCC are rarely reported.

The aim of this study was to investigate the mechanism of MSCs favoring HCC metastasis in inflammatory microenvironment. We first demonstrated that the metastasis of human HCC cell lines was strengthened by the supernatant of MSCs pretreated with IFNγ and TNFα, and the cell lines were also observed undergoing EMT. Then we found that the combination of IFNγ and TNFα would provoke the expression of TGFβ in MSCs and the enhancement of metastasis and EMT of HCC cell lines showed as a TGFβ dependent manner. Furthermore, we employed SSEA-4 as a surface marker to identify MSCs in clinical HCC tissues. The results illustrated that overexpression of SSEA-4 was found to be significantly correlated with poor prognosis of HCC. EMT of HCC lead by MSCs in inflammatory microenvironment was also associated with a shorter cancer-free interval (CFI) and a worse overall survival (OS). Our results suggest that the MSCs in tumor inflammatory microenvironment may be elicited of overexpression of TGFβ, which will promote EMT of HCC that lead to tumor metastasis.

## Results

### MSCs Pretreated by Proinflammatory Cytokines Promote the Metastasis of HCC Cell

**Figure 1:**
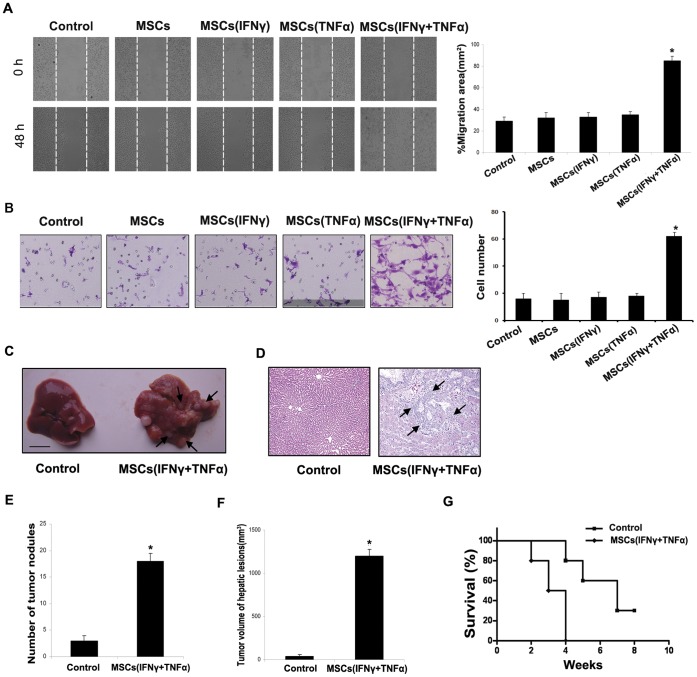
MSCs pretreated by proinflammatory cytokines promote the metastasis of HCC cell. (A) The wound healing assay was employed to determine the migration of SMMC-7721 cells, cells were monitored every 24 h for 48 h to determine the rate of migration into the scratched area (*P<0.05; ×200). (B) Invasiveness of cells was determined using Transwell assay. Cells were co-cultured with MSCs after being stimulated by IFNγ and TNFα, and then plated in the upper chamber of the Transwell and allowed to grow for 24 h in serum-free medium, 5% fetal bovine serum was placed in the lower chamber. Number of cells that invaded through the Matrigel was counted in 10 fields under the ×20 objective lens (*P<0.05; ×200). (C) Pictures of metastatic liver nodules in nude mice by splenic-vein injection of SMMC-7721 cells. The arrows indicate the metastatic tumor on the surface of the liver. (D) H&E staining was performed on serial sections of metastatic tumors and normal liver (×100), the arrow indicate the metastatic tumor in the liver issue; The number (E) and the volume (F) of nodules were quantified on livers of nude mice (n = 10 per group) 6 weeks after splenic vein injection of SMMC-7721 cells co-cultured with MSC after being stimulated by IFNγ and TNFα. Values for individual mice are shown above the bars. (G) Survival rate of nude mice 6 weeks after splenic vein injection of different treated cells (Control was SMMC-7721 cells group, *P<0.05).

To investigate whether MSCs could promote the HCC metastasis in inflammatory microenvironment, we examined the effect of MSCs stimulated by both IFNγ and TNFα on cell motility by a scratch wound-healing assay. As was shown in Figure 1A, SMMC-7721 cells that exposed to conditioned medium of MSCs after being stimulated by both IFNγ and TNFα exhibited significantly enhanced mobility compared with other control cells. By Matrigel invasion assay, an increase in cell invasion was observed in SMMC-7721 cells after being co-cultured with conditioned medium of MSCs which were stimulated by both IFNγ and TNFα in comparison with other control groups (Fig. 1B). The SMMC-7721 cell was replaced by Hep-3B to repeat the experiments mentioned above, the same results were obtained (Fig. S1A, S1B).

We also detected the capacity of MSCs that being stimulated by both IFNγ and TNFα on SMMC-7721 metastasis *in vivo* by splenic vein metastasis assay. A significant increase in hepatic metastasis was noted in mice receiving injection of SMMC-7721 cells co-cultured with conditioned medium of MSCs after being stimulated by both IFNγ and TNFα versus control group (Fig. 1C-F), and mice survival rate was also effected (Fig. 1G). A similar pulmonary metastasis was observed in the tail vein injection model (Fig. S2). These results indicated that MSCs that were stimulated by IFN-γ and TNF-α could effectively enhance the metastatic and invasive ability of HCC cells.

### MSCs Pretreated by Proinflammatory Cytokines Lead Epithelial-mesenchymal Transition of HCC Cells

**Figure 2:**
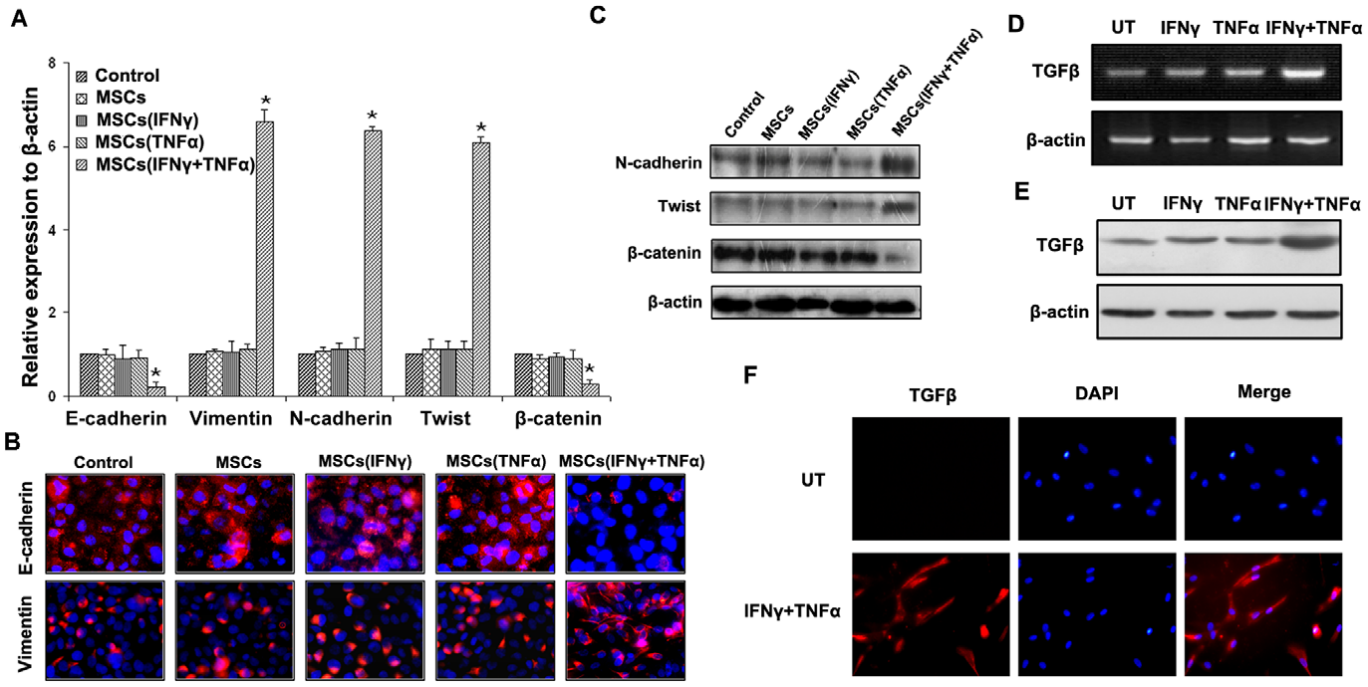
MSCs pretreated by proinflammatory cytokines lead EMT of HCC cells and up-regulation of TGF-β in MSCs. (A) qPCR was used to detect changes in expression of EMT genes in SMMC-7721 HCC cells following co-culture with MSC after being stimulated by IFNγ and TNFα. Results presented represent mean ± SEM (n = 3); (B) Immunofluorescent staining of E-cadherin and Vimentin was performed in SMMC-7721 cells, nuclei were counterstained with DAPI (×200); (C) Western blot was used to detect the expression of N-cadherin, Twist and β-catenin in SMMC-7721 cells. PCR (D) and western-blot (E) were used to detected TGFβ expression in MSCs, and the results showed that TGFβ was overexpressed in MSCs stimulated by IFNγ and TNFα both on mRNA and protein levels. (F) Immunofluorescent staining was used to confirm that up-regulation of TGFβ in MSCs stimulated by combination of IFNγ and TNFα (×200).

Because of some results indicating the critical association between the metastasis and EMT [16], we probed the EMT markers in SMMC-7721 cells that exposed to conditioned medium of MSCs after being stimulated by both IFNγ and TNFα. As was shown in Figure 2A, MSCs, which were pretreated by both IFNγ and TNFα, promoted SMMC-7721 cells exhibiting the typical EMT markers, including down-regulation of epithelial markers E-cadherin and β-catenin and up-regulation of mesenchymal markers Vimentin, N-cadherin and Twist. The cells co-cultured with MSCs stimulated by IFNγ or TNFα, respectively, did not exhibit the typical EMT markers, as well as the control group (*P*<0.05). The result of qPCR was confirmed by immunofluorescent staining and western blot (Fig. 2B, 2C). The same phenomenon was observed in Hep-3B cells co-cultured with conditioned medium of MSCs after being stimulated by both IFNγ and TNFα (Fig. S1C, S1D). Taken together, our results suggested that MSCs pretreated by both IFNγ and TNFα could induce EMT of HCC cells *in vitro*.

### Proinflammatory Cytokines Lead to Up-regulation of TGF-β in MSCs

There were many studies suggested that TGFβ was playing an important role in the induction of EMT. We used TGFβ (1 ng/ml) to stimulate SMMC-7721 cells for 48 h, and the results showed that TGFβ not only induced EMT in SMMC-7721 cells, but also promoted cells motility and invasive abilities (Fig. S3). We thus examined TGFβ expression in MSCs as a molecular response to IFNγ and TNFα treatment. The results showed that combination of IFNγ and TNFα could up-regulate TGFβ expression in MSCs not only in mRNA but also in protein levels (Fig. 2D, 2E), compared with control groups. ELISA results showed that the concentration of TGFβ in MSCs stimulated by IFNγ and TNFα was 1.76±0.14 ng/ml, which was enough to induce cell EMT. Immunofluorescence results further confirmed that overexpression of TGFβ was observed in MSCs stimulated by IFNγ and TNFα (Fig. 2F).

### TGFβ Depletion in MSCs Reverses the Metastasis and EMT of HCC Cells Induced by MSCs in Inflammation Microenvironment

To understand the contribution of TGFβ in MSCs on the metastasis and EMT of HCC, we employed siRNA to inhibit the expression of TGFβ in MSCs and characterized the cells for their induction of HCC cells metastasis and EMT. As was shown in Figure S4, the transfection efficiency of TGFβ siRNA into MSCs was more than 70%, and the expression of TGFβ in MSCs was not up-regulated even they were treated with IFNγ and TNFα.

**Figure 3:**
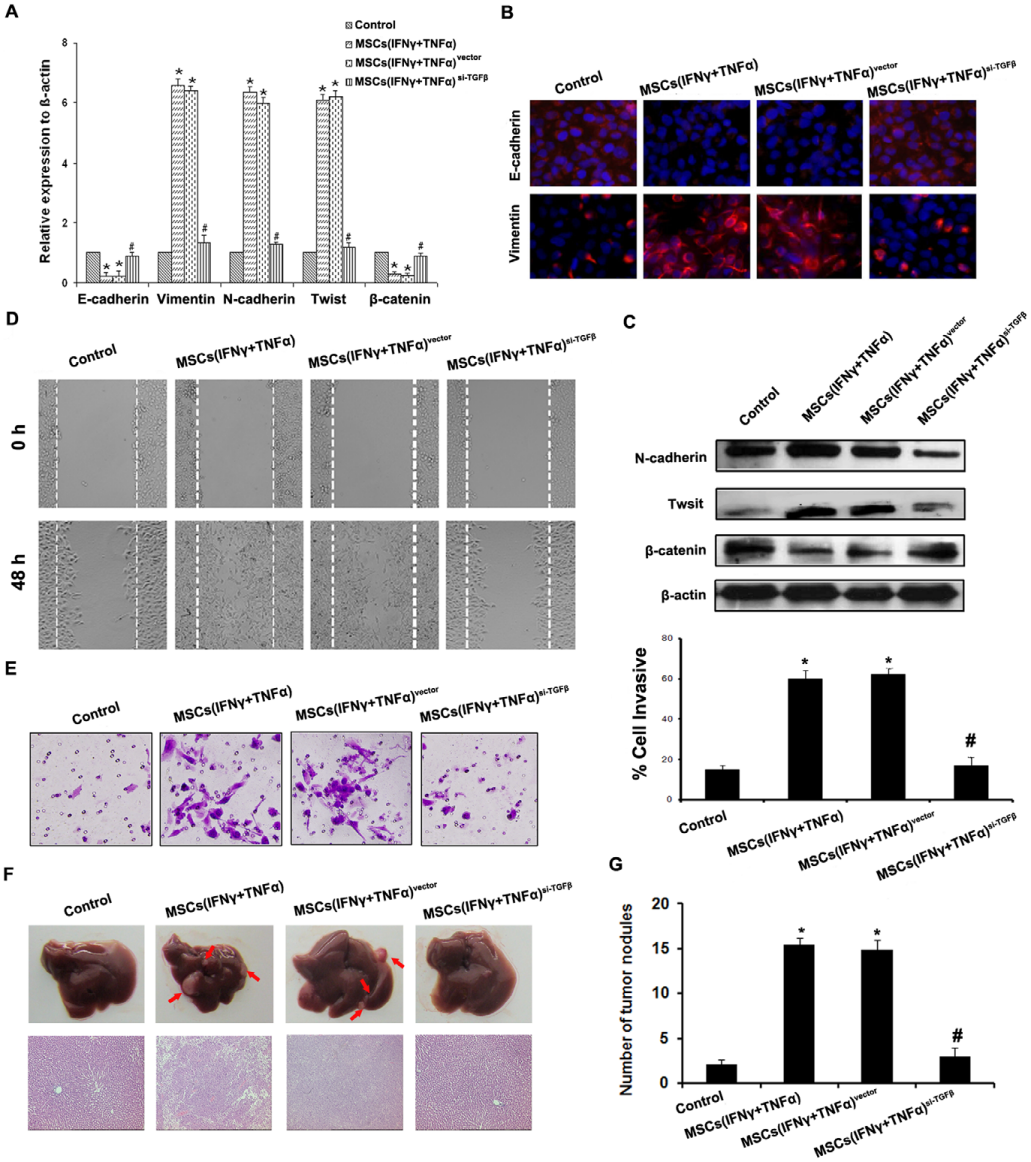
TGF-β depletion in MSCs reverses the promotive effect on metastasis and EMT of HCC cells induced by MSCs in inflammation microenvironment. (A) Expression of EMT genes was detected by qPCR (normalized to β-actin); (B) E-cadherin and Vimentin expression was performed by immunofluorescent staining, nuclei were counterstained with DAPI (×200). (C) Western blot was used to detect the expression of N-cadherin, Twist and β-catenin, SMMC-7721 cells co-cultured with MSCs^si-TGFβ^ stimulated by both IFNγ and TNFα did not present EMT; (D) The wound healing assay was employed to determine the migration of SMMC-7721 cells (×200); (E) Invasiveness of SMMC-7721 cells was determined using Transwell assay; (F) The metastatic liver nodules in nude mice by splenic-vein injection of SMMC-7721 cells. The arrows indicate the metastatic tumor on the surface of the liver (upper). H&E staining was performed on serial sections of metastatic tumors and normal liver (bottom, ×100); (G) The number of nodules were quantified on livers of nude mice (n = 10 per group) 6 weeks after splenic vein injection of SMMC-7721 cells. (*P<0.05 versus Control group; #*P*<0.05 versus MSCs(IFNγ+TNFα) and MSCs(IFNγ+TNFα)^vector^; ×200).

We then examined effect of TGFβ knockdown MSCs on metastasis and EMT of HCC cells in inflammation microenvironment. As a result, TGFβ knockdown in MSCs led to up-regulate E-cadherin and β-catenin, and down-regulate Vimentin, N-cadherin and Twist of SMMC-7721 cells and Hep-3B cells compared with control (Fig. 3A, 3B, 3C and Fig. S5A, S5B), which finally reduced the cell motility and invasive abilities *in vitro* and *in vivo* (Fig. 3D, 3E, 3F, 3G and Fig. S5C, S5D). All these data indicated that the enhancement of metastasis and EMT of HCC cell lines was associated with the up-regulation of TGFβ by MSCs in inflammation microenvironment.

### MSCs Present in HCC Inflammation Microenvironment

**Figure 4:**
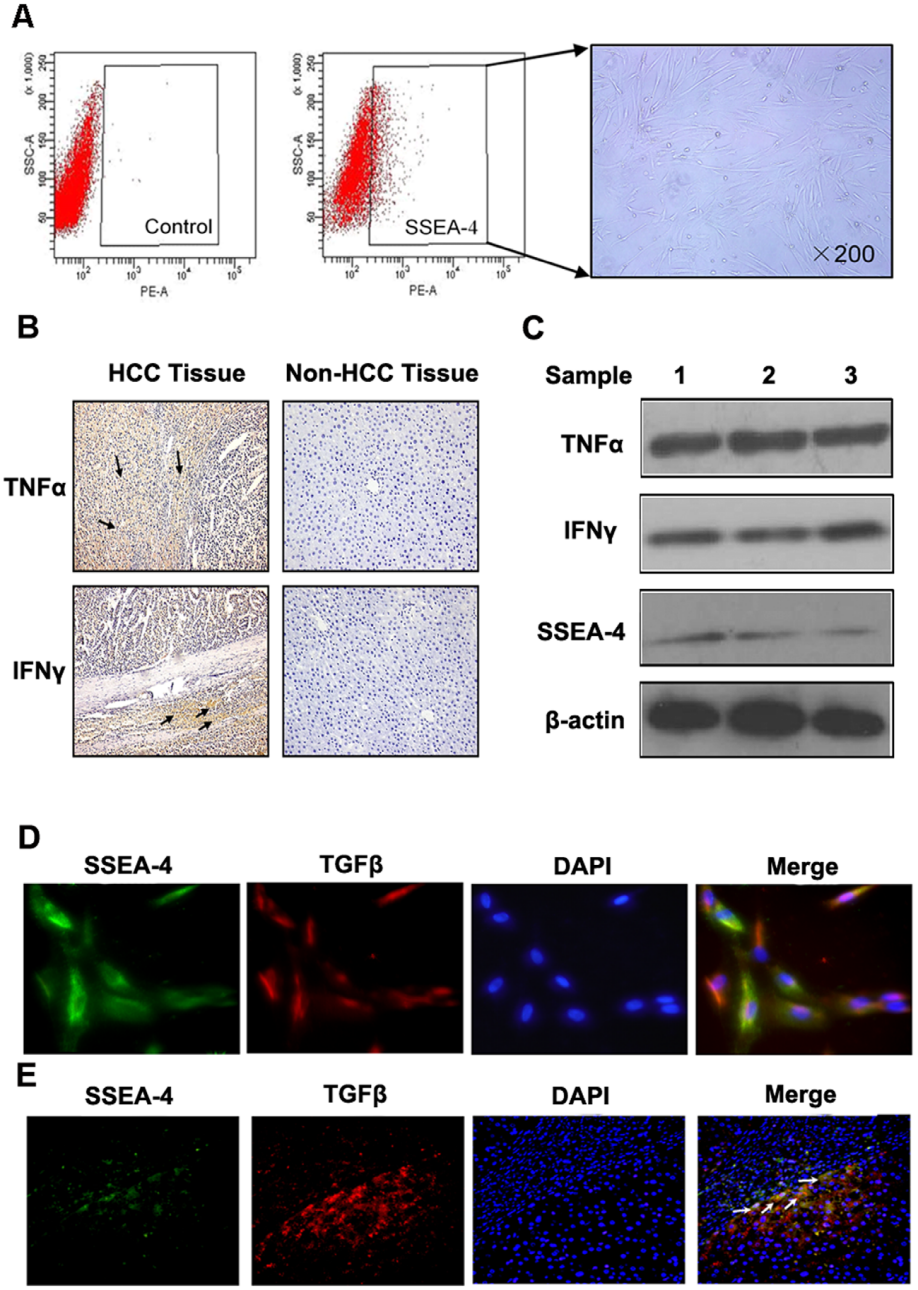
MSCs present in HCC inflammation microenvironment. (A) FACS was used to isolate SSEA-4^+^ cells from fresh HCC tissues, and negative control was showed at left, SSEA-4^+^ cells adhered to the plastic present a homogeneous cell-monolayer characteristic of MSCs (right); (B) In 10 cases of HCC and normal liver tissues, IHC were employed to detect expression of IFNγ and TNFα (×200). (C) The expression of TNF-α, IFN-γ and SSEA4 respectively was detected in three samples of fresh HCC tissues by using western-blot. (D) Double immunofluorescent staining was used to confirm that TGFβ and SSEA-4 expression in MSCs stimulated by combination of IFNγ and TNFα (×400); (E) Double immunofluorescent staining was used to confirm that TGFβ and SSEA-4 expression in HCC tissue (×200).

SSEA-4 was reported as a useful marker for the isolation of MSCs from human bone marrow [20], and we confirmed the result mentioned above by SSEA-4 immunofluorescence staining on human bone marrow-derived MSCs (Fig. S6A). We detected the SSEA-4 expression in fresh human HCC tissue by FACS and the results demonstrated that the percentage of SSEA-4 positive cell was about 5.6±0.9%. We also found that SSEA-4^+^ cells that sorted from human HCC tissues could adhere to the plastic and produce a homogeneous cell-monolayer characteristic of MSCs (Fig. 4A). Expanded cells were subjected to their surface phenotype CD34^−^, CD45^−^, HLR-DR^−^, CD19^−^, CD90^+^, CD105^+^, CD105^+^, FIK1^+^ (Fig. S6B), which was in agreement with those previously described for MSCs [21]. Additional characterization of expanded cells was also performed, which confirmed their ability to differentiate into adipocytes and osteoblasts (Fig. S6C). All these results confirmed that SSEA-4 could be an effective marker to identify MSCs in HCC tissues.

In addition, we examined the inflammatory factors IFNγ and TNFα in HCC, 10 cases of HCC and normal liver tissues were measured by immunohistochemistry. The results showed that the expression of IFNγ and TNFα in HCC tissues, especially at the edge of tumor, was higher than in normal liver tissues (Fig. 4B). Three samples of fresh human HCC tissues were selected to detect the expression of TNF-α, IFN-γ and SSEA4 respectively. In each HCC tissue, there was TNF-α, IFN-γ and SSEA4 expression (Fig. 4C). Taken the results together, we concluded that MSCs really existed in HCC inflammation microenvironment.

Double immunofluorescent staining was used to confirm that TGFβ and SSEA-4 expression MSCs stimulated by combination of IFNγ and TNFα and in HCC tissue, and the result showed that red fluorescence existed around green fluorescence at the edge of tumor or tumor stromal indicating that MSCs in HCC inflammation microenvironment would up-regulate TGFβ expression (Fig. 4D, 4E).

### Correlations of MSCs with Clinicopathologic Characteristics of HCC

**Figure 5:**
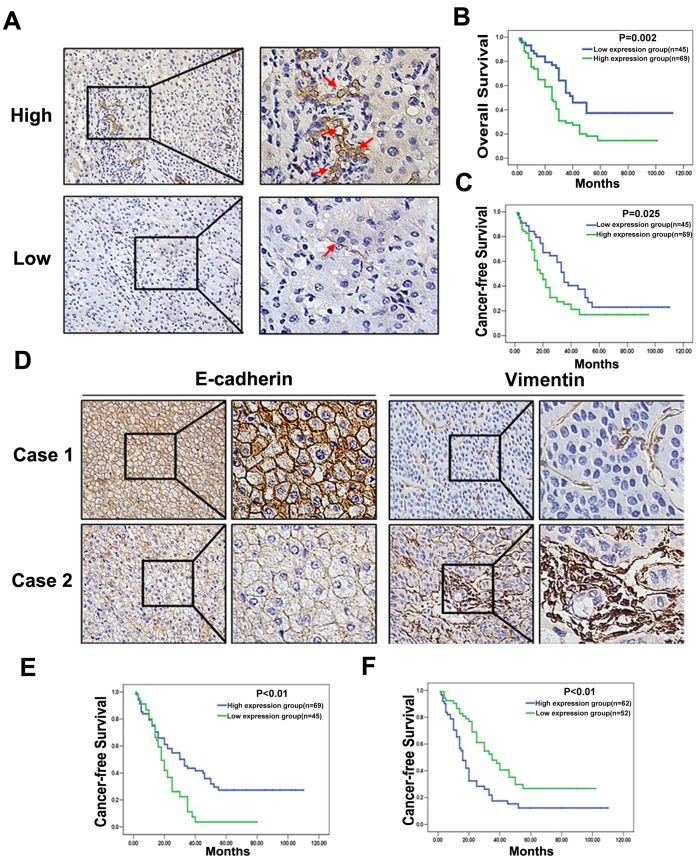
Correlations of MSCs and EMT markers expression with Clinicopathologic Characteristics of HCC. (A) SSEA-4 expression was seen high in 69/114 of HCC tissues (upper left×200, upper right×400), and low in 45/114 (bottom left×200, bottom right×400); (B) Estimated overall survival according to the expression of SSEA-4 in 114 cases of HCCs (the Kaplan-Meier method). Log-rank test shows that HCC patients in the high SSEA-4 expression group have poorer overall survival than those in the low SSEA-4 expression group (*P* = 0.002). (C) Cancer-free survival was analyzed in the same cohort of HCC patients and the results showed that HCC patients in the high SSEA-4 expression group also have poorer cancer-free survival than those in the low SSEA-4 expression group (*P* = 0.025). (D) Immunohistochemistry of E-cadherin and Vimentin in two representative cases without (Case 1) or with (Case 2) EMT change. Membranous expression of E-cadherin was down-regulated and cytoplasmic translocation of Vimentin was up-regulated in case with EMT change (×200); (E) Kaplan-Meier survival analysis of CFI in HCC cases with preserved versus down-regulated E-cadherin expression; (F) Kaplan-Meier survival analysis of CFI in HCC cases with preserved versus up-regulated Vimentin expression.

MSCs were detected by SSEA-4 expression using IHC. Interestingly, SSEA-4 expression was often observed at the edge of the tumor or the surrounding stromal tissue where was infiltrated with much inflammatory cells (Fig. 5A, upper panel). High expression of MSCs was detected in 60.5% of HCC tissues (69/114). The SSEA-4 expression level was found to be significantly higher in HCC patients with Cirrhosis (P = 0.009), Multiple nodules (P = 0.010), Portal vein thrombosis (P = 0.016), Tumor margin (P = 0.017), and UICC TNM (P = 0.012) stage, BCLC stage (P = 0.005) (Table 1). According to the SSEA-4 IHC results, all 114 HCC patients were divided into two groups: the high expression group (n = 69) and low expression group (n = 45) (Fig. 5A). HCC patients with high expression of SSEA-4 group had either worse OS (median survival time, 29.39 months versus 40.24 months, P = 0.002; Fig. 5B) or worse CFI (median disease-free survival time, 24.39 months versus 34.80 months, P = 0.025; Fig. 5C) than those within low expression of SSEA-4.

**Table 1:** Correlations Between SSEA-4 Expression and Clinicopathologic Variables of HCC.

Clinicopathologic Parameters	N	SSEA4 expression levels		*P*-Value
		Low	High	
**Age (y)**				
≤60	81	31	50	0.681
>60	33	14	19	
**Gender**				
Male	91	35	56	0.660
Female	23	10	13	
**Cirrhosis**				
Presence	88	29	59	0.009*
Absence	26	16	10	
**BCLC stage**				
A	43	24	19	0.005*
B or C	71	21	50	
**Tumor size (cm)**				
≤3	51	22	29	0.472
>3	63	23	40	
**Tumor margin**				
Clear	36	20	16	0.017*
Invovled	78	25	53	
**Tumor nodule number**				
Solitary	36	7	29	0.010*
Multiple (≥2)	78	38	40	
**Portal vein thrombosis**				
Presence	64	19	45	0.016*
Absence	50	26	24	
**UICC TNM stage**				
T1–2	47	24	23	0.012*
T3–4	67	21	46	
**Edmondson grade**				
Low(I/II)	45	22	23	0.204
High(III/IV)	69	23	46	

(*P<0.05).

### Analysis of EMT Markers Expression in Human HCC Tissues and Prognosis of HCC with MSCs

Due to the location of MSCs, TGFβ in HCC tissue expression was up-regulated around MSCs at the edge of tumor or tumor stromal (Fig. 4E). IHC was performed to investigate the expression of EMT markers in HCC patients. Among HCC tissues, 34.2% of the samples showed preserved expression of E-cadherin (Case 1 of Fig. 5D, Left panel), which was the hallmark of EMT, decreased E-cadherin expression (Case 2 of Fig. 5D, Left panel) was identified in 65.8% of the samples. Survival analysis demonstrated a significant decrease in CFI in patients with down-regulation of E-cadherin (Fig. 5E). A higher incidence of Vimentin overexpression (Case 2 of Fig. 5D, Right panel) was shown in HCC tissues (41.2% versus 58.8%), and overexpression of Vimentin was associated with a shorter CFI (Fig. 5F). By multivariate Cox regression analysis, MSCs in HCC tissues were associated with EMT, the markers of which were down-regulation of E-cadherin and overexpression of Vimentin, and factors indicating clinical aggressiveness (e.g. Portal vein thrombosis; Table 2). The results suggested that the mechanisms that MSCs in inflammatory microenviroment promoting HCC cells metastasis by inducing EMT via overexpression of TGF-β might also exist in HCC specimens.

**Table 2:** Multivariate Analysis by a Cox Proportional Hazards Regression Model.

Variables	N	Univariate Analysis		Multivariate Analysis	
		RR (95% CI)	*P* Value	RR (95% CI)	*P* Value
**Age (y)**					
≤60	81	1	0.983	1	0.124
>60	33	1.000(0.988–1.013)		0.988(0.972–1.003)	
**Gender**					
Male	91	1	0.422	1	0.302
Female	23	0.807(0.478–1.362)		0.739(0.416–1.313)	
**Cirrhosis**					
Presence	88	1	0.639	1	0.006*
Absence	26	0.886(0.535–1.468)		0.448(0.251–0.798)	
**Tumor size (cm)**					
≤3	51	1	0.451	1	0.316
>3	63	0.844(0.544–1.311)		1.285(0.787–2.101)	
**Tumor margin**					
Clear	39	1	0.537	1	0.372
Invovled	75	0.862(0.539–1.380)		0.783(0.457–1.340)	
**Tumor nodule number**					
Solitary	36	1	<0.001*	1	0.024*
Multiple (≥2)	78	2.335(1.453–3.754)		1.921(1.092–3.381)	
**Portal vein thrombosis**					
Presence	64	1	<0.001*	1	0.016*
Absence	50	2.332(1.469–3.702)		1.929(1.132–3.286)	
**UICC TNM stage**					
T1–2	47	1	0.001*	1	0.020*
T3–4	67	0.459(0.291–0.726)		0.540(0.321–0.908)	
**Edmondson grade**					
Low(I/II)	45	1	0. 110	1	0.093
High(III/IV)	69	0.529(0.327–0.856)		0.613(0.347–1.085)	
**SSEA-4 expression**					
Low	45	1	0.031*	1	0.042*
High	69	0.605(0.383–0.955)		0.514(0.273–0.967)	
**E-cadherin expression**			0.001*		
High	39	1		1	0.003*
Low	75	2.247(1.404–3.597)		1.782(1.021–3.110)	
**Vimentin expression**					
Low	47	1	0.001*	1	0.039*
High	67	0.469(0.300–0.734)		0.444(0.258–0.763)	

(*P<0.05).

## Discussion

MSCs play an important role in treating various degenerative diseases and immune disorders. They have great potential for correcting aberrant immune reactions. Previous studies showed that these cells could be expanded and induced ex vivo, terminally differentiate into osteoblasts, chondrocytes, adipocytes, myotubes, neural cells and hematopoietic supporting stroma [1], [2], [22]. Taking advantage of homing capacities to the primary tumor site, MSCs have been used for the targeted delivery of immunostimulatory cytokines and chemokines, suicide genes, growth-factor antagonists, and oncolytic viruses after systemic administration [23].

Recent evidence suggests that MSCs participate in tumor growth and metastasis, partially due to their immunosuppressive and pro-angiogenic properties. MSCs stimulate human osteosarcoma tumor-bearing nude mice tumor growth and increase osteosarcoma cell proliferation in the presence of MSC-conditioned media [24]; Subcutaneously implanted human mammary carcinomas co-injected with MSCs acquire an increased metastatic potential [17]. In our study, we used IFNγ and TNFα stimulating human MSCs from bone marrow to imitate the tumor inflammatory microenvironment, and then co-cultured with different HCC cells. The results demonstrated thatthe metastasis of human HCC cell lines was strengthened by the supernatant of MSCs which were pretreated with IFNγ and TNFα, and the HCC cell lines also were observed undergoing EMT, which was associated with HCC metastasis. Similar *in vivo* results were also observed in nude mice injected with SMMC-7721 cells after being co-cultured with MSCs stimulated by combination of IFNγ and TNFα. To explore the potential mechanisms involved in the function of MSCs, we investigated whether EMT induction by MSCs was through TGFβ, which is known to provoke EMT in many types of epithelial cells in culture [25] and generally considered one of the master positive regulators of EMT. As expected, IFNγ and TNFα significantly up-regulated the expression of TGFβ in MSCs. All of these results clearly demonstrated that the EMT induced by MSCs in inflammation microenvironment was an important mechanism underlying HCC development and metastasis. However, Li *et al*. found that the MSC enhanced tumor growth but significantly inhibited the invasiveness and metastasis of HCC, possibly through down-regulation of TGFβ in HCC cells [26]. Niess H, *et al.* demonstrated that homing of systemcially injected MSCs to growing HCC xenografts with concomitant activation of CCL5 or Tie2 promoters delivered the tumor-specific expression of suicide gene *HSC-Tk*. Combined with GCV, the administration of *CCL5/HSV-TK^+^* MSCs led to significant reduction in tumor mass [27]. We considered that the paradoxical results mentioned above might due to the factors dealt with MSCs were different. In our study, we considered the influnce of inflammatory microenvironment on MSCs, and used IFNγ and TNFα stimulating MSCs to imitate the tumor inflammatory microenvironment, and then took the conditioned medium to treat different HCC cells, which was different from the conditions mentioned aboved. The results showed that MSCs stimulated by combination of IFNγ and TNFα secreted TGFβ and then promoted HCC cells metastasis. Due to the different treatment of MSCs, the effect of MSCs on the growth and metastasis of HCC cells is controversial. At present, the identification of MSC was typically negative for the hematopoietic markers CD34, CD45, and positive for the surface markers CD29, CD73, CD90, CD105, CD106 and STRO-1. Recently, Eun J. Gang, *et al.* used SSEA-4, a marker previously thought to be specific to human embryonic stem cells and very early cleavage to blastocyst stage embryos, to isolate MSCs from whole human bone marrow [20]. In this study, we first used SSEA-4 to isolate MSCs from HCC tissues, and furture characterized the surface markers as well as the differentiation abilities into adipocytes or osteoblasts, the results showed that SSEA-4^+^ cells in HCC tissues were just MSCs from human bone marrow and SSEA-4 could be used for the prospective isolation of MSCs in tumor tissues.

From 114 cases of human HCC tissues samples, we investigated the correlation of MSCs with clinicopathologic characteristics of HCC patients. In HCC inflammation microenvironment, MSCs detected by SSEA-4 expression turned out to be significantly correlated with multiple nodules, Cirrhosis, Tumor margin, and Portal vein thrombosis of HCC, which were widely accepted markers for metastasis and poor prognosis of HCC [28]. The Kaplan-Meier analysis indicated that HCC patients with high SSEA-4 expression in general had worse prognosis than those with low SSEA-4 expression. A multivariate Cox regression analysis indicated that high SSEA-4 expression was an independent risk factor for the prognosis of HCC patients, suggesting that MSCs may be a useful prognostic biomarker of HCC.

In order to further investigate whether MSCs in inflammation microenvironment were exerting their effects through induction of EMT, a number of specific genetic markers of EMT were examined in HCC tissues. E-cadherin present in most epithelial cells is a calcium-dependent transmembrane glycoprotein. Vimentin has been consistently associated with mesenchymal transition in epithelial cells [29], [30]. E-cadherin down-regulation and Vimentin up-regulation are commonly observed in more invasive basal cancer subtypes and have been positively correlated with poor prognosis in breast cancer patients [17]. Interestingly, our results showed that HCC undergoing EMT, which labeled by down-regulation of E-cadherin and up-regulation of Vimentin, were associated with a shorter CFI and a worse OS. Further investigations demonstrated that induction of EMT was correlated with SSEA-4 expression in HCC tissues, which revealed that the major channel of MSCs in inflammation microenvironment increased HCC metastatic potential may be EMT induction, the multivariate Cox regression analysis indicated that both MSCs and EMT induction could predict HCC metastasis and recurrence.

In conclusion, our study showed that MSCs recruited to HCC microenvironment were isolated by SSEA-4 and significantly correlated with a poor prognosis of HCC. Furthermore, we demonstrated the critical role of EMT in metastasis of HCC induced by MSCs in inflammation microenvironment. These results elaborated on the major mechanisms involved in HCC metastasis and provided MSCs as prediction of prognosis and identification of new treatment targets for future HCC management.

## Materials and Methods

### Cell Lines

Human MSCs (a gift from Institute of Health Sciences and Shanghai Institute of Immunology, Chinese Academy of Sciences, Shanghai, China; ref. [31]) were cultured in Dulbecco’s modified Eagle’s medium (DMEM) nutrient mix F12 with fetal bovine serum (FBS, 10%; Invitrogen). HCC cell lines including SMMC-7721 cells (from Chinese Academy Cell Bank in 2001) and Hep-3B cells (from the American Type Culture Collection in 2008) were cultured in DMEM with 10% FBS at 37°C in a humidified atmosphere containing 5% CO_2_.

### Patients and Tissue Specimens

The study protocol was approved by the Ethics Committee of Eastern Hepatobiliary Surgery Hospital (Approved Code: EHBHKY2012-002-7, the Verification of Ethics Committee was in the supporting information) and prior informed consent was obtained (One of the written consent was in the supporting information). Specimens of HCC tissues were obtained from 114 HCC patients who underwent hepatic resection at Eastern Hepatobiliary Surgery Hospital of Second Military Medical University. These patients, from February 1998 to December 2006, included 91 males and 23 females with a median age of 52 years (range: 11–87), and all of the specimens were subjected to immunohistochemisty (IHC). In addition, 10 cases fresh specimens of normal liver tissues from hemangioma excision and 10 cases fresh specimens of HCC were collected for fluorescence activated cell sorting (FACS) and IHC.

### The Conditioned Medium

MSCs were stimulated with by IFNγ (20 ng/ml) and TNFα (20 ng/ml) for 12 h, then the culture medium of which was abandoned and replaced with fresh DMEM nutrient mix F12 with 10% FBS. After being continue cultured for 24 h, the conditioned medium was obtained by collection and 0.22 µm filtration of the supernatant media from MSCs.

### Wound Healing and Transwell Assay

The methods for wound healing and the Transwell assay have been described [18], [32], [33]. For migration assay, wound-healing assay was done. Cells (1×10^5^) were seeded on 24-well dish and incubated for 24 h, monolayer was then disrupted with a cell scraper (1.2 mm width), and photographs were taken at 0 and 48 h in a phase-contrast microscope. Experiments were carried out in triplicate, and four fields of each point were recorded. For Transwell assay, Boyden chambers (8 µm pore size) were coated with 200 µl Matrigel at 200 µg/ml and incubated overnight. Cells (1×10^5^) in medium without serum were plated in the upper chamber, and the medium containing 5% FBS was added in the lower chamber as a chemoattractant. After 24 h of incubation at 37°C, the cells were fixed in 4% formaldehyde and stained with crystal violet dye, and the cells that invaded through the pores to the lower surface of the filter were counted under a microscope. Three invasion chambers were used per condition. The values obtained were calculated by averaging the total number of cells from three filters.

### Nude Mouse Splenic Vein Metastasis Assay and Tail Vein Metastasis Assay

All procedures involving animals were performed in accordance with the institutional animal welfare guidelines of Second Military Medical University and approved by the Ethics Committee of Eastern Hepatobiliary Surgery Hospital (Approved Code: EHBHKY2012-002-7). Cells were injected into the splenic vein or the tail vein of 8-week-old nude mice (BALB/c strain) at 1×10^6^ cells/injection site. The mice were sacrificed after 6 weeks and the number and volume of metastatic tumors were assessed. Tumor size was measured by use of a caliper and volume was calculated as length×height×width×0.5236 with reference to a previous report [18].

### Quantitative Real-time Polymerase Chain Reaction (qPCR)

Total RNA extraction, complementary DNA (cDNA) synthesis, and qPCR were performed as described [19]. The primer sequences used in qPCR were shown in Table S1.

### Western-blot Analysis

Total soluble proteins extraction from cultivated cells and western-blot analysis were performed as described [19]. Antibodies used in western-blot experiments were specific for either TGFβ (Abcam) or β-actin (Invitrogen) and goat anti-rabbit secondary antibody (Invitrogen).

### Immunofluorescence

About 10^4^ cells were seeded on a 48-well dish. After 24 h, the cells were washed with phosphate-buffered saline (PBS) twice and fixed in 4% paraformaldehyde and 0.1% Triton X 100 in PBS buffer at 4°C for 30 minutes. After being washed with PBS, the cells were incubated with the blocking solution (10% goat serum in PBS), and then incubated with the primary antibodies for overnight, washed with PBS, and finally incubated with secondary antibodies (Invitrogen) at 37°C for 2 h. After stained with DAPI, all matched samples were photographed using immunofluorescence microscope and identical exposure times.

### Short Interfering RNA (siRNA) Synthesis and Transient Transfection

Three siRNA sequences of TGFβ were designed by using Oligoengine software and confirmed by nucleotide BLAST searches. The three putative candidate sequences and a scrambled sequences with no significant homology were listed in Table S1. Transfections were performed using a Lipofectamine 2000 kit (Invitrogen) according to the manufacturer’s instructions. Cells (1–3×10^6^) grew to a confluency of 50%–60% in 10 cm petri dishes were transfected with siRNA sequence or their relative mock sequences, then the cells were observed under fluorescence microscope and harvested 48 hours after transfection.

### FACS Analysis

The fresh specimens of HCC and normal liver tissues was transferred to a petri dish, where the tissue was gently minced and filtered (100 mm) to remove large aggregates, the cell suspension was filtered (40 mm) and nonparenchymal cells were separated by discontinuous density gradients of Percoll (Pharmacia Biotech). The SSEA-4 antibody (eBioscience) was added to the final cell suspension at 0.1 µg/10^6^ cells and incubated at 4°C for 30 minutes before washing with blocking buffer, then stained cells were analyzed on a FACS (Becton Dickinson, San Jose, CA). In the sorting experiments, cells were purified based on the expression of SSEA-4 (positives and negatives). For clonal analysis, SSEA-4^+^ cells were deposited into single wells of a 96-well dish.

Wells with single cell colonies were harvested and expanded into clonal cell lines. PE-conjugated antihuman antibody to CD105 and CD34 as well as FITC-conjugated antihuman CD45 and CD90 (eBioscience) were applied for characterization of human MSCs.

### IHC Analysis

The sample processing and IHC procedures were performed as described [18]. The antibodies used in IHC are listed in Table S2. All IHC staining was independently scored by three experienced specialists. The score for SSEA-4 expression was based on intensity of staining and the percentage of positive cells (0 = none, 1 = 1%–5%, 2 = 6%–50%, 3 = 51%–100%), the expression of SSEA-4 in HCC specimens was divided into a low expression group (0 or 1) and a high expression group (2 or 3). The expression of E-cadherin and Vimentin was scored as reduced, preserved and overexpression, as described [34], reduced expression of E-cadherin was interpreted as down-regulation of E-cadherin, and overexpression of Vimentin was interpreted as up-regulation. The expression of IFNγ and TNFα was graded from 0 to 3 (0 = no staining; 1 = 1%–25%; 2 = 26%–50%; 3≥50%) with only 3 considered a positive IHC result.

### Statistical Analysis

All data, expressed as mean ± SEM, were from at least three separate experiments. Groups were compared by analysis of variance (ANOVA) with a posteriori contrast by least significant difference; or by Student t-test using the Microsoft Excel Analysis ToolPak (Microsoft, Redmond, WA). P<0.05 was considered significant.

## Supporting Information

Figure S1 **MSC stimulated by IFNγ and TNFα induced Hep-3B cells migration, invasion and EMT** ***in vitro*** **.** (A) The wound healing assay was employed to determine the migration of in Hep-3B cells, cells were monitored every 24 h for 2 days to determine the rate of migration into the scratched area. The results showed that closure of in Hep-3B HCC cells following co-culture with MSC after being stimulated by IFNγ and TNFα as significantly nearer than that of in Hep-3B cell lines cultured in the same medium that had not been exposed to MSCs or MSCs treated with IFNγ, TNFα respectively (*P<0.05; ×200); (B) Invasiveness of cells was determined using Transwell assay. Cells were co-cultured with MSCs after being stimulated by IFNγ and TNFα, and then plated in the upper chamber of the Transwell and allowed to grow for 24 hours in serum-free medium, 5% fetal bovine serum was placed in the lower chamber. Number of cells that invaded through the Matrigel was counted in 10 fields under the ×20 objective lens. Our data showed that the percent of cell invasion following co-culture with MSC stimulated by IFNγ and TNFα was also up-regulated (*P<0.05; ×200). (C) qPCR was used to detected changes in expression of EMT genes in Hep-3B HCC cells following co-culture with MSC stimulated by IFNγ and TNFα, the control represents the level of expression in in Hep-3B cell lines cultured in the same medium that had not been exposed to MSCs. Results presented represent mean of triplicate experiments ± SEM; (D) Immunofluorescent staining of E-cadherin and Vimentin was performed in in Hep-3B cells, nuclei were counterstained with DAPI (×200);
(TIF)

Figure S2 **The pulmonary metastasis in the tail vein injection model.** (A) Pictures of metastatic lung nodules in nude mice by tail-vein injection of SMMC-7721 cells. The arrows indicate the metastatic tumor on the surface of the lung. (B) H&E staining was performed on serial sections of metastatic tumors and normal lung (×200); (C) and (D) The number and the volume of nodules were quantified on lung of nude mice (n = 10 per group) 6 weeks after tail vein injection of SMMC-7721 cells co-cultured with MSC after being stimulated by IFNγ and TNFα. Values for individual mice are shown above the bars.
(TIF)

Figure S3 **TGFβ induced EMT in SMMC-7721 cells and promoted cells motility, invasive abilities** ***in vitro*** **.** (A) qPCR was used to detected changes in expression of EMT genes in SMMC-7721 HCC cells stimulated by TGFβ (1 ng/ml) for 48 h, and the results showed that TGFβ induced EMT in SMMC-7721 cells obviously. Results presented represent mean of triplicate experiments ± SEM; (B) The wound healing assay was employed to determine the migration of in SMMC-7721 cells, cells were monitored every 24 h for 2 days to determine the rate of migration into the scratched area. The results showed that closure of in SMMC-7721 cells stimulated by TGFβ as significantly nearer than that of in control group (*P<0.05; ×200); (C) Invasiveness of cells was determined using Transwell assay. Cells stimulated by TGFβ (1 ng/ml) for 48 h, and then plated in the upper chamber of the Transwell and allowed to grow for 24 h in serum-free medium, 5% fetal bovine serum was placed in the lower chamber. Number of cells that invaded through the Matrigel was counted in 10 fields under the ×20 objective lens. Our data showed that the percent of cell invasion stimulated by TGFβ was also up-regulated (*P<0.05; ×200).
(TIF)

Figure S4 **Suppression of TGFβ expression in MSCs by siRNA.** (A) Transfections of TGFβ siRNA into MSCs were performed using a Lipofectamine, and FAM was observed under fluorescence microscope (Right, ×400); (B) The transfection efficiency of TGFβ siRNA was detected by FACS was more than 70%; (C) RT-PCR and (D) Western-blot results showed that the expression of TGFβ was markedly decreased in MSCs^si-TGFβ^ stimulated by both IFNγ and TNFα, the inhibitory efficiency was more than 80% compared with the MSCs^vector^ stimulated by both IFNγ and TNFα.
(TIF)

Figure S5 **Silencing TGFβ inhibited the motility, invasion abilities of Hep-3B cells induced by MSCs stimulated by IFNγ and TNFα** ***in vitro.*** (A) Expression of EMT genes was detected by qPCR (normalized to β-actin); (B) E-cadherin and Vimentin expression was performed by immunofluorescent staining, nuclei were counterstained with DAPI. Hep-3B cells co-cultured with MSCs^si-TGFβ^ stimulated by both IFNγ and TNFα did not present EMT (×200); (C) The wound healing assay was employed to determine the migration of Hep-3B cells (×200); (D) Invasiveness of Hep-3B cells was determined using Transwell assay. The motility and invasion abilities of Hep-3B cells induced by MSCs stimulated by both IFNγ and TNFα were reversed accompany with TGFβ depletion (*P<0.05 versus Control group; #*P*<0.05 versus MSCs(IFNγ+TNFα) and MSCs(IFNγ+TNFα)^vector^; ×200).
(TIF)

Figure S6 **Identification of MSCs in HCC inflammation microenvironment.** (A) Immunofluorescence was used to identify human MSCs from bone marrow presenting SSEA-4 expression (×400); (B) The surface phenotype of isolated cells at passage 3 was detected by FACS, and CD34^−^, CD45^−^, HLR-DR^−^, CD19^−^, CD90^+^, CD105^+^, CD29^+^, FIK1^+^were in agreement with surface phenotype of MSCs; (C) Multilineage differentiation of isolated cells at passage 5 for adipocytes was demonstrated by Oil Red O staining (Left, ×200), and for osteoblasts showed by von kossa staining (Right, ×200).
(TIF)

Table S1 **Sequence of the oligonucleotides for real-time PCR and siRNA construct-making assays.** The primer sequences used in qPCR for E-cadherin, Vimentin, N-cadherin, Twist, β-catenin, TGFβ, β-actin, and three siRNA sequences of TGFβ and a scrambled sequences with no significant homology were listed in Table S1.
(DOC)

Table S2 **List of proteins tested by antibodies and characteristics of the corresponding antibodies used.** The antibodies used in the western-blot, immunofluorescence or immunohistochemistry for SSEA-4, E-cadherin, Vimentin, IFNγ, TNFα, and TGFβ.
(DOC)
