# Supplementary figures and images for: Mesenchymal Stem Cells in Inflammation Microenvironment Accelerates Hepatocellular Carcinoma Metastasis by Inducing Epithelial-Mesenchymal Transition
Source: PLoS One. 2012 Aug 28;7(8):e43272. doi: 10.1371/journal.pone.0043272 (PMC3429457; doi:10.1371/journal.pone.0043272)

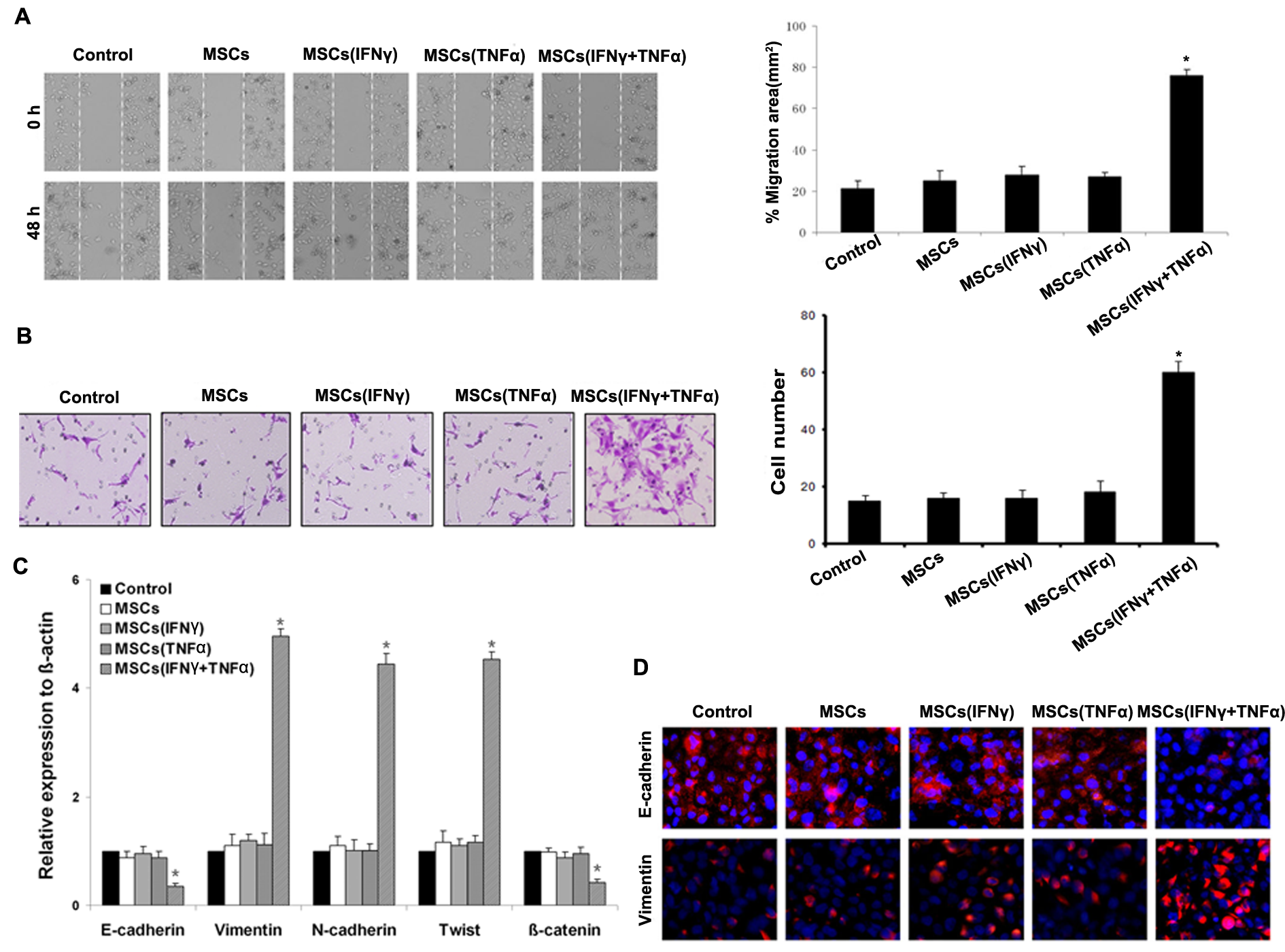

Supplement: Figure S1 — MSC stimulated by IFNγ and TNFα induced Hep-3B cells migration, invasion and EMT in vitro . (A) The wound healing assay was employed to determine the migration of in Hep-3B cells, cells were monitored every 24 h for 2 days to determine the rate of migration into the scratched area. The results showed that closure of in Hep-3B HCC cells following co-culture with MSC after being stimulated by IFNγ and TNFα as significantly nearer than that of in Hep-3B cell lines cultured in the same medium that had not been exposed to MSCs or MSCs treated with IFNγ, TNFα respectively (*P<0.05; ×200); (B) Invasiveness of cells was determined using Transwell assay. Cells were co-cultured with MSCs after being stimulated by IFNγ and TNFα, and then plated in the upper chamber of the Transwell and allowed to grow for 24 hours in serum-free medium, 5% fetal bovine serum was placed in the lower chamber. Number of cells that invaded through the Matrigel was counted in 10 fields under the ×20 objective lens. Our data showed that the percent of cell invasion following co-culture with MSC stimulated by IFNγ and TNFα was also up-regulated (*P<0.05; ×200). (C) qPCR was used to detected changes in expression of EMT genes in Hep-3B HCC cells following co-culture with MSC stimulated by IFNγ and TNFα, the control represents the level of expression in in Hep-3B cell lines cultured in the same medium that had not been exposed to MSCs. Results presented represent mean of triplicate experiments ± SEM; (D) Immunofluorescent staining of E-cadherin and Vimentin was performed in in Hep-3B cells, nuclei were counterstained with DAPI (×200); (TIF) [file pone.0043272.s001.tif]

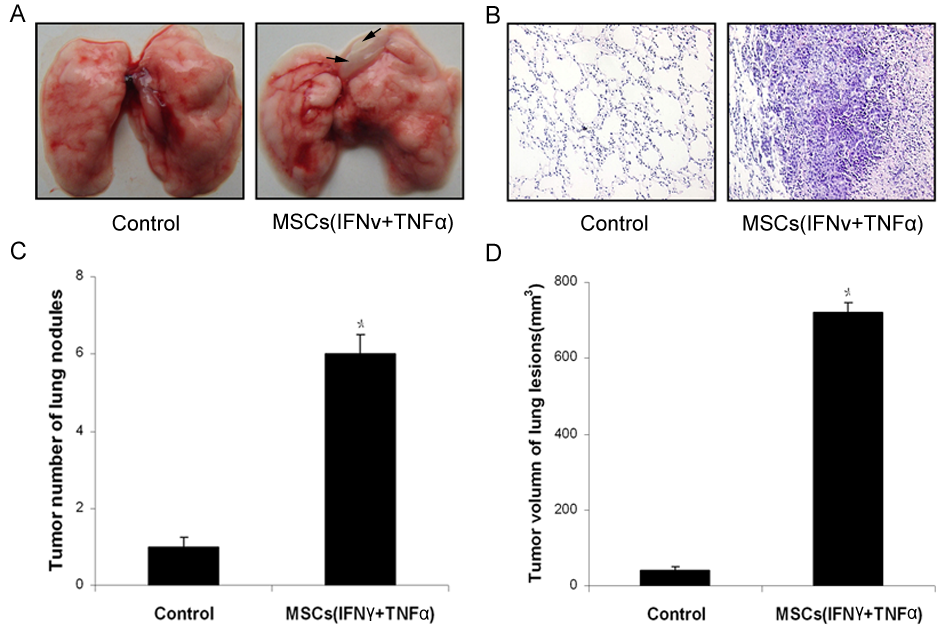

Supplement: Figure S2 — The pulmonary metastasis in the tail vein injection model. (A) Pictures of metastatic lung nodules in nude mice by tail-vein injection of SMMC-7721 cells. The arrows indicate the metastatic tumor on the surface of the lung. (B) H&E staining was performed on serial sections of metastatic tumors and normal lung (×200); (C) and (D) The number and the volume of nodules were quantified on lung of nude mice (n = 10 per group) 6 weeks after tail vein injection of SMMC-7721 cells co-cultured with MSC after being stimulated by IFNγ and TNFα. Values for individual mice are shown above the bars. (TIF) [file pone.0043272.s002.tif]

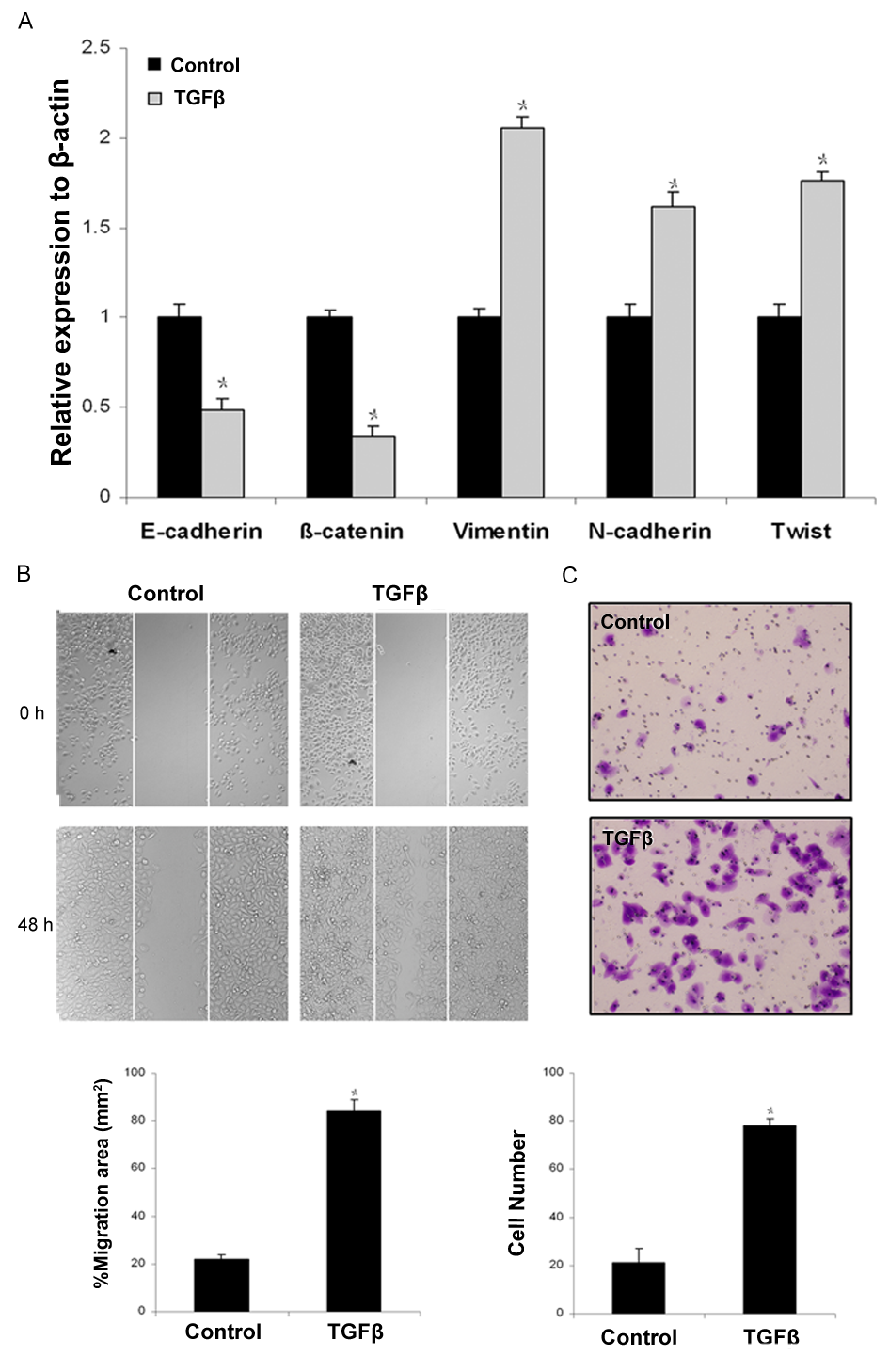

Supplement: Figure S3 — TGFβ induced EMT in SMMC-7721 cells and promoted cells motility, invasive abilities in vitro . (A) qPCR was used to detected changes in expression of EMT genes in SMMC-7721 HCC cells stimulated by TGFβ (1 ng/ml) for 48 h, and the results showed that TGFβ induced EMT in SMMC-7721 cells obviously. Results presented represent mean of triplicate experiments ± SEM; (B) The wound healing assay was employed to determine the migration of in SMMC-7721 cells, cells were monitored every 24 h for 2 days to determine the rate of migration into the scratched area. The results showed that closure of in SMMC-7721 cells stimulated by TGFβ as significantly nearer than that of in control group (*P<0.05; ×200); (C) Invasiveness of cells was determined using Transwell assay. Cells stimulated by TGFβ (1 ng/ml) for 48 h, and then plated in the upper chamber of the Transwell and allowed to grow for 24 h in serum-free medium, 5% fetal bovine serum was placed in the lower chamber. Number of cells that invaded through the Matrigel was counted in 10 fields under the ×20 objective lens. Our data showed that the percent of cell invasion stimulated by TGFβ was also up-regulated (*P<0.05; ×200). (TIF) [file pone.0043272.s003.tif]

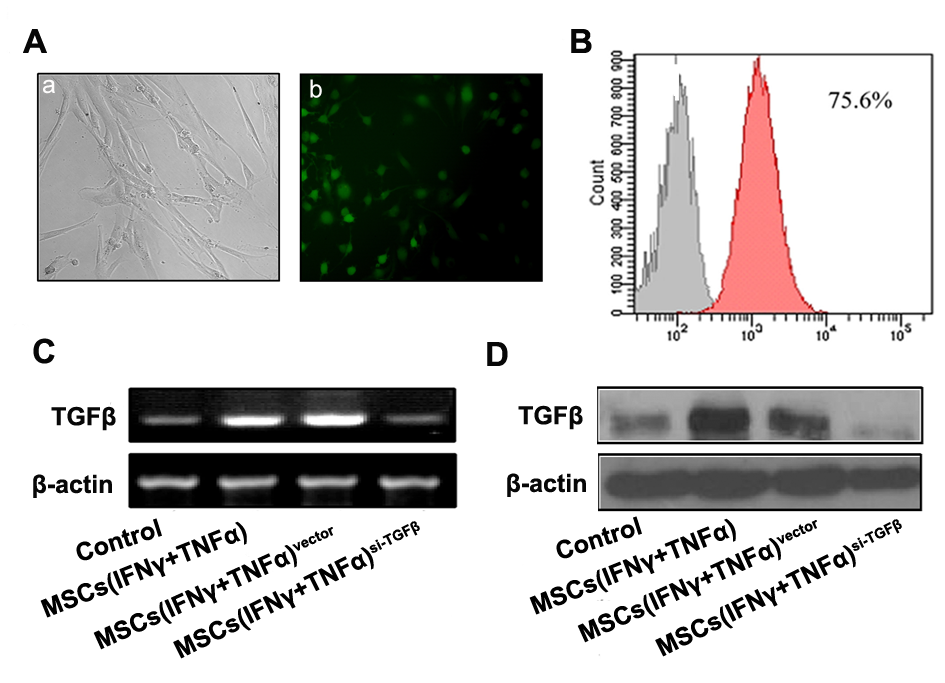

Supplement: Figure S4 — Suppression of TGFβ expression in MSCs by siRNA. (A) Transfections of TGFβ siRNA into MSCs were performed using a Lipofectamine, and FAM was observed under fluorescence microscope (Right, ×400); (B) The transfection efficiency of TGFβ siRNA was detected by FACS was more than 70%; (C) RT-PCR and (D) Western-blot results showed that the expression of TGFβ was markedly decreased in MSCssi-TGFβ stimulated by both IFNγ and TNFα, the inhibitory efficiency was more than 80% compared with the MSCsvector stimulated by both IFNγ and TNFα. (TIF) [file pone.0043272.s004.tif]

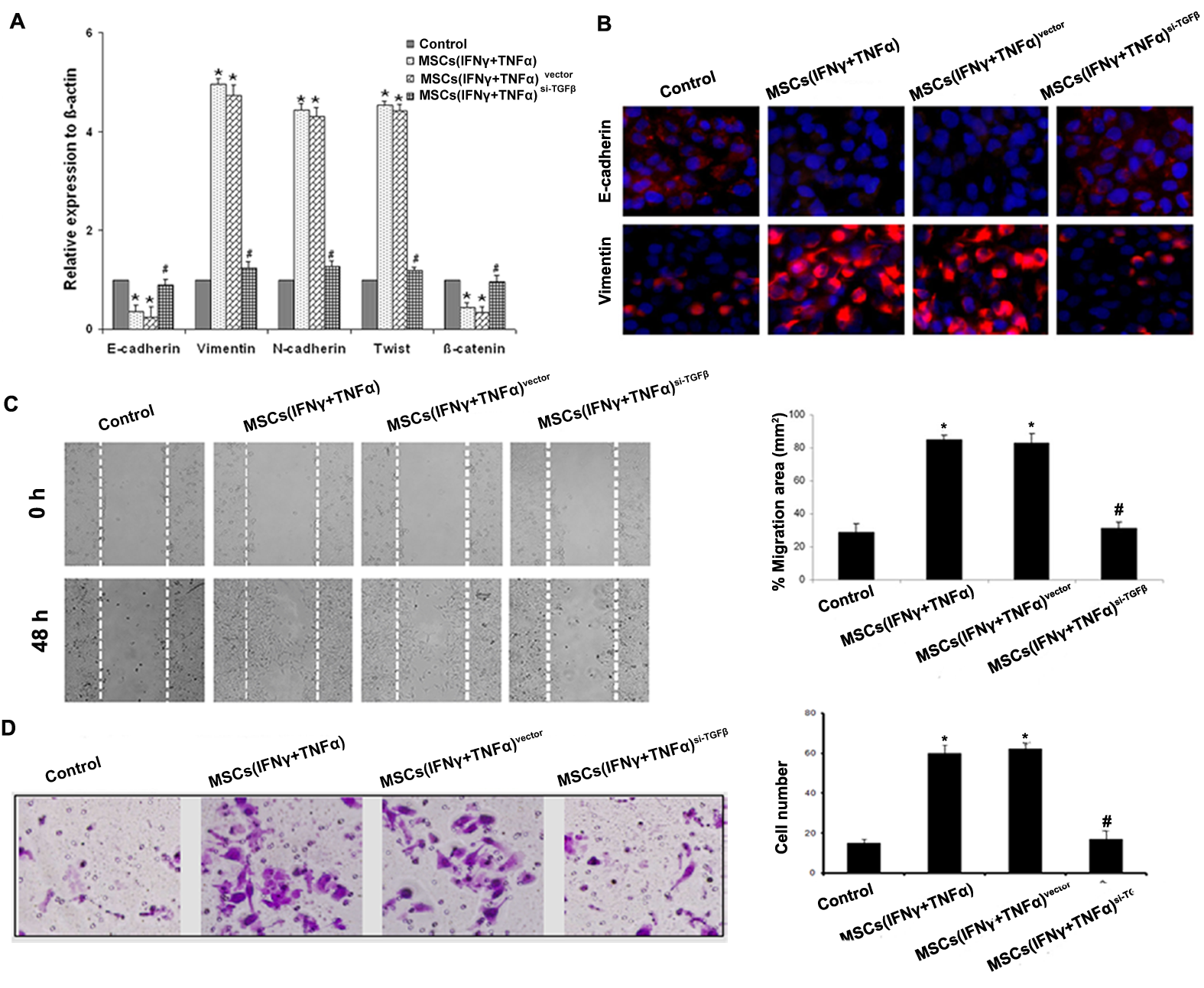

Supplement: Figure S5 — Silencing TGFβ inhibited the motility, invasion abilities of Hep-3B cells induced by MSCs stimulated by IFNγ and TNFα in vitro. (A) Expression of EMT genes was detected by qPCR (normalized to β-actin); (B) E-cadherin and Vimentin expression was performed by immunofluorescent staining, nuclei were counterstained with DAPI. Hep-3B cells co-cultured with MSCssi-TGFβ stimulated by both IFNγ and TNFα did not present EMT (×200); (C) The wound healing assay was employed to determine the migration of Hep-3B cells (×200); (D) Invasiveness of Hep-3B cells was determined using Transwell assay. The motility and invasion abilities of Hep-3B cells induced by MSCs stimulated by both IFNγ and TNFα were reversed accompany with TGFβ depletion (*P<0.05 versus Control group; #P<0.05 versus MSCs(IFNγ+TNFα) and MSCs(IFNγ+TNFα)vector; ×200). (TIF) [file pone.0043272.s005.tif]

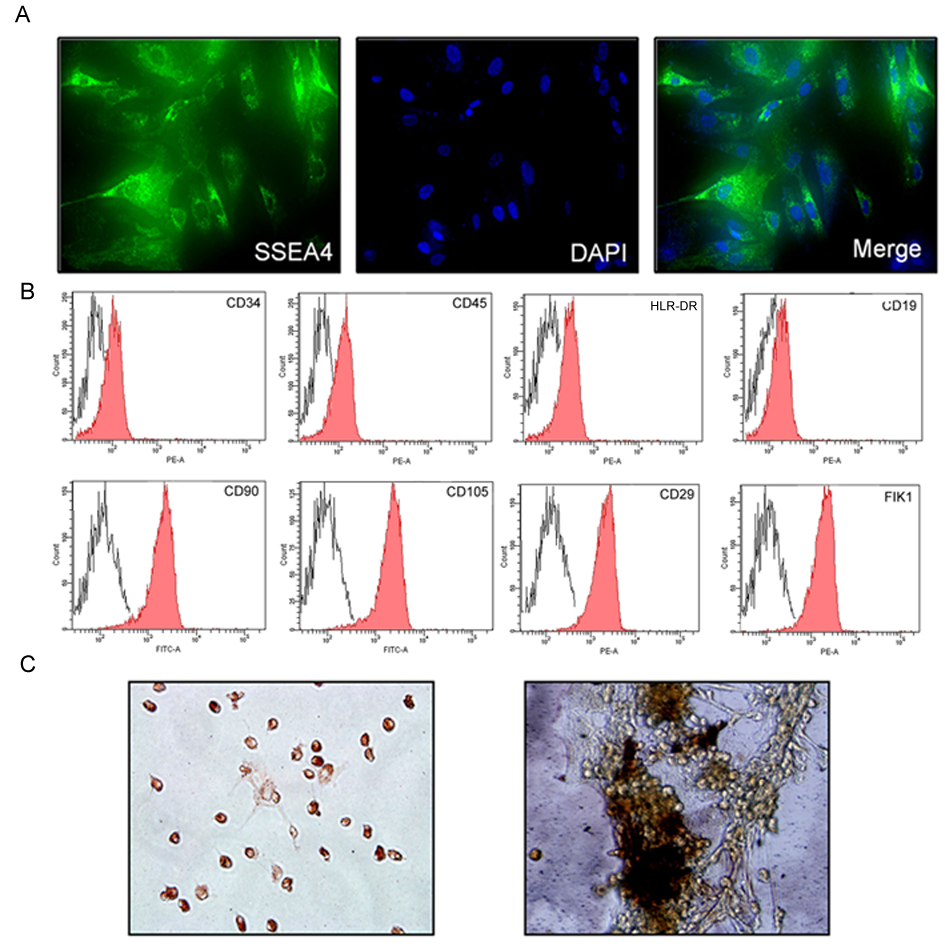

Supplement: Figure S6 — Identification of MSCs in HCC inflammation microenvironment. (A) Immunofluorescence was used to identify human MSCs from bone marrow presenting SSEA-4 expression (×400); (B) The surface phenotype of isolated cells at passage 3 was detected by FACS, and CD34−, CD45−, HLR-DR−, CD19−, CD90+, CD105+, CD29+, FIK1+were in agreement with surface phenotype of MSCs; (C) Multilineage differentiation of isolated cells at passage 5 for adipocytes was demonstrated by Oil Red O staining (Left, ×200), and for osteoblasts showed by von kossa staining (Right, ×200). (TIF) [file pone.0043272.s006.tif]
