# Supplementary material for: Mesenchymal Stem Cells in Inflammation Microenvironment Accelerates Hepatocellular Carcinoma Metastasis by Inducing Epithelial-Mesenchymal Transition
Source: PLoS One. 2012 Aug 28;7(8):e43272. doi: 10.1371/journal.pone.0043272 (PMC3429457; doi:10.1371/journal.pone.0043272)
Supplement: Table S1 — Sequence of the oligonucleotides for real-time PCR and siRNA construct-making assays. The primer sequences used in qPCR for E-cadherin, Vimentin, N-cadherin, Twist, β-catenin, TGFβ, β-actin, and three siRNA sequences of TGFβ and a scrambled sequences with no significant homology were listed in Table S1. (DOC) [file pone.0043272.s007.doc]

**Table S1. Sequence of the oligonucleotides for real-time PCR and siRNA construct-making assays**

| **Assays** | **Gene** | **Sequence (5’ 🡪 3’)** | |
| --- | --- | --- | --- |
| **Real-time PCR** | E-cadherin | F | TGAAGGTGACAGAGCCTCTGGA |
|  |  | R | TGGGTGAATTCGGGCTTGTT |
|  | Vimentin | F | TGGCCGACGCCATCAACACC |
|  |  | R | CACCTCGACGCGGGCTTTGT |
|  | N-cadherin | F | GCGCGTGAAGGTTTGCCAGTG |
|  |  | R | CCGGCGTTTCATCCATACCACAA |
|  | Twist | F | GGTCCATGTCCGCGTCCCACTAG |
|  |  | R | CGCCCCACGCCCTGTTTCTT |
|  | β-catenin | F | AGCCGACACCAAGAAGCAGAGATG |
|  |  | R | CGGCGCTGGGTATCCTGATGT |
|  | TGFβ | F | GCCGAGCCCTGGACACCAAC |
|  |  | R | GCGCCCGGGTTATGCTGGTT |
|  | β-actin | F | CTCCATCCTGGCCTCGCTGT |
|  |  | R | GCTGTCACCTTCACCGTTCC |
| **TGFβ siRNA** | Sequence 1 | Sense | CACUGCAAGUGGACAUCAATT |
|  |  | Antisense | UUGAUGUCCACUUGCAGUGTT |
|  | Sequence 2 | Sense | GCAAGACUAUCGACAUGGATT |
|  |  | Antisense | UCCAUGUCGAUAGUCUUGCTT |
|  | Sequence 3 | Sense | GCAUAUAUAUGUUCUUCAATT |
|  |  | Antisense | UUGAAGAACAUAUAUAUGCTT |
|  | Control | Sense | UUCUCCGAACGUGUCACGUTT |
|  |  | Antisense | ACGUGACACGUUCGGAGAATT |
