# Supplementary material for: Mesenchymal Stem Cells in Inflammation Microenvironment Accelerates Hepatocellular Carcinoma Metastasis by Inducing Epithelial-Mesenchymal Transition
Source: PLoS One. 2012 Aug 28;7(8):e43272. doi: 10.1371/journal.pone.0043272 (PMC3429457; doi:10.1371/journal.pone.0043272)
Supplement: Table S2 — List of proteins tested by antibodies and characteristics of the corresponding antibodies used. The antibodies used in the western-blot, immunofluorescence or immunohistochemistry for SSEA-4, E-cadherin, Vimentin, IFNγ, TNFα, and TGFβ. (DOC) [file pone.0043272.s008.doc]

**Table S2. List of proteins tested by antibodies and characteristics of the corresponding antibodies used**

| **Protein** | **Assay** | **Antibody** | **Origin** | **Concentration** |
| --- | --- | --- | --- | --- |
| SSEA-4 | IHC/IF | #4755, Cell Signaling Technology | mouse | 1:1000 |
| E-cadherin | IHC/IF | Ab 11512, Abcam | rabbit | 1:1000 |
| Vimentin | IHC/IF | Ab 8545, Abcam | rabbit | 1:500 |
| IFNγ | IHC | 15521, Sigma | mouse | 25μg/ml |
| TNFα | IHC | T6817, Sigma | mouse | 25μg/ml |
| TGFβ | IF | Ab66043, Abcam | rabbit | 5μg/ml |
| TGFβ | WB | Ab66043, Abcam | rabbit | 1μg/ml |

Abbreviations: IHC: immunohistochemistry; IF: immunofluorescence; WB: Western blot.
